# The Proposal of Molecular Mechanisms of Weak Organic Acids Intake-Induced Improvement of Insulin Resistance in Diabetes Mellitus via Elevation of Interstitial Fluid pH

**DOI:** 10.3390/ijms19103244

**Published:** 2018-10-19

**Authors:** Yoshinori Marunaka

**Affiliations:** 1 Research Institute for Clinical Physiology, Kyoto Industrial Health Association, Kyoto 604-8472, Japan; marunaka@koto.kpu-m.ac.jp; Tel.: +81-75-802-0131 (ext. 2322); 2 Research Center for Drug Discovery and Pharmaceutical Development Science, Research Organization of Science and Technology, Ritsumeikan University, Kusatsu 525-8577, Japan; 3 Department of Molecular Cell Physiology, Graduate School of Medical Science, Kyoto Prefectural University of Medicine, Kyoto 602-8566, Japan; 4 Japan Institute for Food Education and Health, St. Agnes’ University, Kyoto 602-8013, Japan

**Keywords:** weak organic acid, food, pH, interstitial fluid, insulin, binding affinity, alkalization

## Abstract

Blood contains powerful pH-buffering molecules such as hemoglobin (Hb) and albumin, while interstitial fluids have little pH-buffering molecules. Thus, even under metabolic disorder conditions except severe cases, arterial blood pH is kept constant within the normal range (7.35~7.45), but the interstitial fluid pH under metabolic disorder conditions becomes lower than the normal level. Insulin resistance is one of the most important key factors in pathogenesis of diabetes mellitus, nevertheless the molecular mechanism of insulin resistance occurrence is still unclear. Our studies indicate that lowered interstitial fluid pH occurs in diabetes mellitus, causing insulin resistance via reduction of the binding affinity of insulin to its receptor. Therefore, the key point for improvement of insulin resistance occurring in diabetes mellitus is development of methods or techniques elevating the lowered interstitial fluid pH. Intake of weak organic acids is found to improve the insulin resistance by elevating the lowered interstitial fluid pH in diabetes mellitus. One of the molecular mechanisms of the pH elevation is that: (1) the carboxyl group (R-COO^−^) but not H^+^ composing weak organic acids in foods is absorbed into the body, and (2) the absorbed the carboxyl group (R-COO^−^) behaves as a pH buffer material, elevating the interstitial fluid pH. On the other hand, high salt intake has been suggested to cause diabetes mellitus; however, the molecular mechanism is unclear. A possible mechanism of high salt intake-caused diabetes mellitus is proposed from a viewpoint of regulation of the interstitial fluid pH: high salt intake lowers the interstitial fluid pH via high production of H^+^ associated with ATP synthesis required for the Na^+^,K^+^-ATPase to extrude the high leveled intracellular Na^+^ caused by high salt intake. This review article introduces the molecular mechanism causing the lowered interstitial fluid pH and insulin resistance in diabetes mellitus, the improvement of insulin resistance via intake of weak organic acid-containing foods, and a proposal mechanism of high salt intake-caused diabetes mellitus.

## 1. Introduction

Some kinds of food habits with high calorie intake result in metabolic syndrome, which would be a precursor for diseases such as type 2 diabetes mellitus, cardiovascular diseases and cancer [1,2]. Furthermore, high salt intake also results in these diseases including type 2 diabetes mellitus [3], although the molecular mechanism is still unclear. One of the most typical, serious phenomena appearing in type 2 diabetes mellitus is insulin resistance, which causes hyperglycemia and results in various types of disorders such as hypertension, vascular dysfunction, hyper-activation of sympathetic nerve, and renal failure [4,5,6,7,8,9,10,11,12]. In particular, hypertension is one of the most typical clinical symptoms for diagnosis of cardiovascular disorders [10,11,12,13,14,15,16,17]. Many studies indicate the molecular mechanisms developing insulin resistance are based on various specific molecules such as cytokines [18,19,20,21,22,23,24,25,26,27,28,29,30,31,32,33,34,35,36,37,38,39,40,41,42,43,44,45,46,47,48,49,50,51,52,53]. This information [18,19,20,21,22,23,24,25,26,27,28,29,30,31,32,33,34,35,36,37,38,39,40,41,42,43,44,45,46,47,48,49,50,51,52,53] is very helpful to understand the molecular mechanism developing insulin resistance; however, it is still unclear how these factors [18,19,20,21,22,23,24,25,26,27,28,29,30,31,32,33,34,35,36,37,38,39,40,41,42,43,44,45,46,47,48,49,50,51,52,53] interactively develop insulin resistance. Insulin resistance results in hyperinsulinemia due to insufficiency of insulin action on glucose uptake in skeletal muscles and fatty tissues [54,55,56,57,58,59,60]. The number of patients suffering from type 2 diabetes mellitus still continuously increases worldwide [61]. Thus, one of the most important points for prevention and treatment of diabetes mellitus is to clarify molecular mechanisms causing insulin resistance. A tremendous number of studies performed in many laboratories have been trying to clarify the molecular mechanisms causing insulin resistance, and develop the treatment, prevention, and early diagnosis of insulin resistance [12,62,63,64,65,66,67]. Nevertheless, at the present time, we have not yet obtained enough knowledge on how insulin resistance develops. Therefore, we should clarify the molecular mechanism causing insulin resistance for development of fundamental treatments and prevention of diabetes mellitus.

Insulin resistance causes continuous hyperglycemia, one of the most major symptoms of type 2 diabetes mellitus, due to poor uptake of glucose into cells such as skeletal muscles, adipocytes and hepatocytes [40,54]. The continuous hyperglycemia stimulates insulin secretion from pancreatic β cells, exhausting pancreatic β cells, associated with development of dysfunction and damage of pancreatic β cells. Continuous hyperglycemia in type 2 diabetes mellitus patients with insulin resistance, in general, irreversibly causes macro- and micro-vascular complications [68]. This develops stroke, myocardial infarction, obstructive arteriosclerosis, renal dysfunction, blindness, dementia, and peripheral neuropathy. International Diabetes Federation (IDF) [61] has reported that the number of people with diabetes mellitus in the worldwide in 2017 is 425 million, and 642 million people would be recognized as diabetes mellitus by 2040. Many researchers develop various types of drugs for treatment of type 2 diabetes mellitus such as sulfonylurea, biguanide, glucosidase inhibitors, thiazolidine, dipeptidyl-peptidase (PPD) IV inhibitors, sodium-glucose cotransporter 2 (SGLT2) inhibitors [61]. However, an extraordinarily large number of people still suffer from type 2 diabetes mellitus. These facts indicate that these drugs are still little effective to fully treat patients suffering from type 2 diabetes mellitus with insulin resistance, although these newly developed drugs are very efficient for treatment of type 2 diabetes mellitus from a viewpoint of reduction of blood glucose level. The reason for relatively little effectiveness of these drugs on treatment of insulin resistance would be due to the concept of drug development. At the present stage the purpose of drug developments is only or mainly to decrease the level of blood glucose or prevent elevation of blood glucose level. An increase in the blood glucose level is not the essential pathogenesis of type 2 diabetes mellitus, but only a result from occurrence of insulin resistance in type 2 diabetes mellitus [69,70]. For example, sulfonylurea stimulates insulin release from pancreatic β cells; biguanide blocks production of glucose from lactate mainly in the liver; glucosidase inhibitors diminish production of glucose by inhibiting glucosidases involved in breaking down of carbohydrates mainly in the intestine; thiazolidine stimulates glucose uptake via stimulation of adiponectin release from adipocytes by acting peroxisome proliferator-activated receptor (PPAR); dipeptidyl-peptidase IV (DPP-4) inhibitors maintain a high level of insulin by blocking brake-down of incretin in the intestine stimulating insulin release from pancreatic β cells; sodium-glucose cotransporter 2 (SGLT2) inhibitors diminish reuptake of glucose in the renal epithelia. In general, unfortunately all drugs are only designed to reduce the blood glucose level by blocking glucose production, glucose reuptake or insulin release, but not to improve fundamentally insulin resistance.

Weak organic acids such as short-chain fatty acids stimulate the secretion of glucagon-like peptide (GLP) 1 that enhances insulin secretion [71]. Fuddu et al. [71] also suggest that short-chain fatty acids (weak organic acids) may improve insulin resistance; however, the molecular mechanism is unclear. On the other hand, intake of high salt is reported to cause diabetes mellitus; however, the molecular mechanism is also unclear.

In this review article, from a novel viewpoint of ‘regulation of interstitial fluid pH’, I introduce the concept on development of insulin resistance in type 2 diabetes mellitus, the preventing method from insulin resistance with intake of foods containing weak organic acids, and a proposal mechanism of pathogenesis of diabetes mellitus caused by high salt intake.

## 2. Variety of the Interstitial Fluid pH

**Figure 1:**
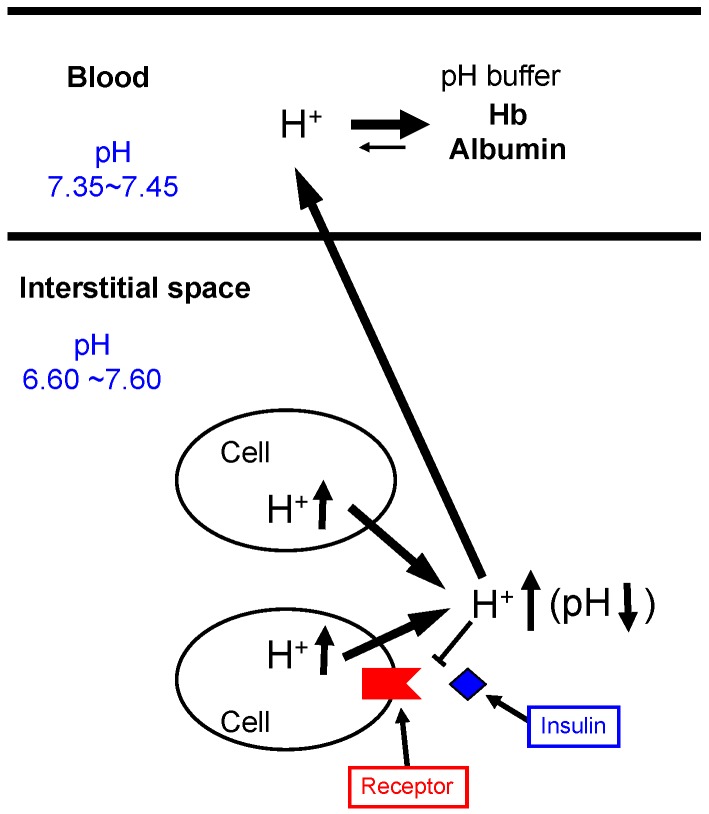
pH of interstitial fluids and blood, and binding affinity of insulin to its receptor. Blood contains very strong, powerful pH-buffering molecules such as hemoglobin (Hb) and albumin, while interstitial fluids have little pH-buffering molecules. Thus, even under metabolic disorder conditions except severe disorders, arterial blood pH is kept constant within the normal range (7.35–7.45), but pH of interstitial fluids becomes lower than the normal level. Modified from Figure 1 in *World J Diabetes* 6(1): 125–135, 2015 [1].

Bodily and cellular functions are controlled by various factors including ions such as Na^+^, K^+^, Ca^2+^, Mg^2+^, Zn^2+^, and Cl^−^ [72,73,74,75,76,77,78,79,80,81,82,83,84,85,86,87,88,89,90,91,92,93,94,95,96,97,98,99,100,101,102,103,104,105,106,107]. On the one hand, pH (−log[H^+^]) is recognized as one of classical items considered to be already fully understood in roles and mechanisms of homeostasis. However, we should pay attention to the interstitial fluid pH, since neurotransmitters and most of hormones show their actions by binding to their receptors facing the interstitial fluid [1,2,108,109]. Thus, changes in the interstitial fluid pH influence the binding affinity of hormones and neurotransmitters to their receptors by modulating the structures of hormones, neurotransmitters and/or their receptors, varying signaling efficiency of hormones and neurotransmitters [1,2,108,109,110]. Among various ionic compositions of the interstitial fluid, pH is a most notable one to be very variable due to much less capacity of pH buffers than that in blood (Figure 1). Unlike the interstitial fluid, pH of arterial blood is well known to be strictly fixed between 7.35 and 7.45 by strong pH-buffering molecules such as hemoglobin (Hb) and albumin existing in blood [1] (Figure 1). The difference of pH-buffering capacity in blood and interstitial fluids indicates that even under metabolically pathophysiological conditions except severe conditions, ‘arterial’ blood pH stays within the normal range (7.35~7.45) [108,109], while pH of interstitial fluids would be changeable out of the normal range [108,109]. It should be noted that hormones and neurotransmitters bind to their receptors facing ‘the interstitial fluids’ but not blood [1] (Figure 1). A further important point is that the conformation of proteins is affected by fluid pH around the proteins, meaning that hormones, neurotransmitters, and their receptors change in their conformations dependent on pH values [111,112]. This conformational alteration caused by pH changes affects the binding affinity of hormones and neurotransmitters to their receptors (Figure 1). Thus, the interstitial fluid pH is one of the most essentially important key factors regulating bodily and cellular functions [111,112]. Nevertheless, we unfortunately pay little attention to interstitial fluid pH and have little information on interstitial fluid pH under pathophysiological conditions.

**Figure 2:**
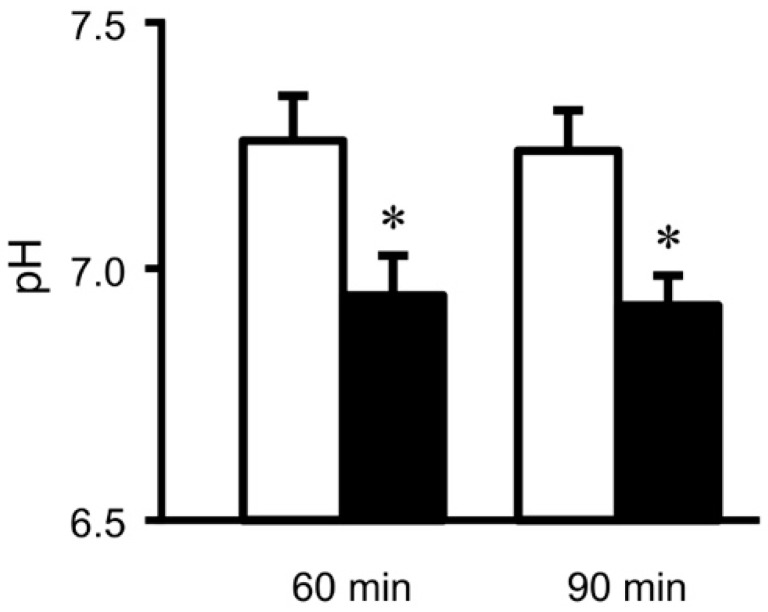
pH of interstitial (extracellular) fluids around the hippocampus of Otsuka Long-Evans Tokushima Fatty (OLETF: a model rat of type 2 diabetes mellitus) and normal (Wistar) rats. The pH value is shown as the mean ± SEM (*n* = 4). The pH values shown in Figure 2 were measured at 60 and 90 min after antimony pH electrodes reached interstitial (extracellular) fluids around the brain hippocampus of OLETF rats (closed columns) and normal (Wistar) rats (open columns). *, *p* < 0.05 compared with that in normal (Wistar) rats at each measured time. Modified from *Mol Cell Therapies* 2:6, 2014 [2].

We should recognize that maintenance of interstitial fluid pH within the normal range is essentially required to keep normal bodily and cellular functions. Indeed, the interstitial fluid pH is lower in diabetes mellitus [2,113,114] (Figure 2). This lowered interstitial fluid pH reduces affinity of insulin to its receptor [110] (Figure 1).

## 3. Physiological Roles of Little pH Buffering Capacity of Interstitial Fluids in Bodily and Cellular Functions

Interstitial fluids provide the place (space) where hormones and neurotransmitters bind to their receptors as the first step to show their actions in our body. Thus, the maintenance of interstitial fluid pH within the normal range is essentially required to keep normal bodily and cellular functions and adapt body conditions to new environments, since abnormal values of the interstitial fluid pH diminish the action of hormones and neurotransmitters [2,113,114,115,116]. Nevertheless, unfortunately unlike blood, interstitial fluids have little pH-buffering molecules, resulting in interstitial fluid pH with much more variable values compared with pH values of arterial blood [108,109]. If the interstitial fluid would contain strong pH-buffering molecules such as albumin, pH of interstitial fluids is very strictly fixed within the normal range (7.35~7.45) like arterial blood pH [108,109]. This pH constancy of the interstitial fluid might be favorable for us to keep our body healthy [108,109]. However, we should also consider the meaning of interstitial pH-buffering molecules from another viewpoint of metabolite movements from peripheral tissues into blood capillary for maintenance of our lives [117,118,119]. The extrusion of metabolites from peripheral tissues requires the driving force for movements into blood capillaries from peripheral tissues via the interstitial fluid [117,118,119]. The driving force of metabolite movements into the blood capillary from the interstitial fluid across the blood capillary wall near veins is maintained by the colloid pressure existing in the blood capillary. The colloid pressure is mainly generated by albumin, a strong pH-buffering molecule. The concentration of albumin in blood capillaries is much higher than that in the interstitial fluid [117,118,119]. If the interstitial fluids would contain strong pH-buffering molecules such as albumin, the interstitial fluid results in having high colloid osmotic pressure. This high colloid osmotic pressure of interstitial fluids diminishes the driving force of metabolite collection into blood capillary [117,118,119,120]. This disturbs collection of metabolites into blood capillaries from interstitial fluids across walls of blood capillaries [117,118,119,120]. Thus, from a viewpoint of pH buffer capacity, the fact of little pH-buffering molecules in interstitial fluids is a weak point to keep ideal activities of hormones and enzymes [108,109]. However, the fact of little pH buffering molecules in the interstitial fluids is a strong point to collect metabolites from peripheral tissues into blood capillaries [117,118,119,120]. Although the pH-buffering capacity of interstitial fluids is much smaller than that of blood, the pH of interstitial fluids is maintained within the normal range due to the extremely large capacity of blood pH-buffering action under the physiological condition [108,109]. Even under pathophysiological conditions with mild metabolic disorders, the pH-buffering capacity in blood is still large enough to maintain pH of arterial blood within the normal range, while the interstitial fluid pH deviates from the normal pH range due to little pH buffer capacity [108,109].

## 4. Sources of H^+^ in Interstitial Fluids

Under normal physiological conditions, the pH of ‘arterial’ blood in mammal is accurately maintained within 7.35~7.45 by strong pH-buffering molecules. However, severe metabolic disorders produce an extremely large amount of acids, which exceed the pH-buffering capacity of arterial blood, resulting in deviation of ‘arterial’ blood pH from the normal range of 7.35~7.45: pH of ‘arterial’ blood <7.35 is defined as acidosis, and pH of ‘arterial’ blood >7.45 is defined as alkalosis. Vomiting of gastric juices, diarrhea (metabolic alkalosis), or hyperventilation (respiratory alkalosis) causes alkalosis, pH of ‘arterial’ blood >7.45. However, alkalosis, in general, occurs transiently but does not chronically occur. Unlike alkalosis, acidosis (especially chronic acidosis) is frequently observed in various metabolic disorders such as diabetes mellitus. Alkalosis and acidosis are well recognized as severe disorders in our body conditions, nevertheless we have little information on the interstitial fluid pH. We could not guarantee that the pH of interstitial fluids really stays within the normal range even under the conditions that the pH of ‘arterial’ blood is normal (7.35~7.45). In fact, the pH of interstitial fluids in type 2 diabetes mellitus is lower than the normal range [2,113,114] even under conditions that the pH of ‘arterial’ blood is kept within the normal range. The pH of interstitial fluids is influenced by the amount of H^+^ provided by glycolysis and organic acids produced in TCA cycle at the ATP synthesis in living cells. Lactic acid, CH_3_-CH(OH)-COOH (CH_3_-CH(OH)-COO^−^ + H^+^), one of typical H^+^ sources, is converted from pyruvic acid, CH_3_-CO-COOH (CH_3_-CO-COO^−^ + H^+^). Lactic acid is a metabolite from anaerobic glycolysis and provides H^+^. For example, skeletal muscles requiring a large amount of energy (ATP) at sudden and excessive exercise produce a tremendous amount of energy, ATP, via an anaerobic glycolytic process converting glucose and glycogen into pyruvic acid. This pyruvic acid is converted to lactic acid under anaerobic conditions, while this pyruvic acid is a substrate for TCA cycle in mitochondria and consumed in TCA cycle under an aerobic condition. This means that under aerobically physiological conditions only little amounts of lactic acid are generated. When TCA cycle functions under an aerobic condition, most of the final product of glycolysis is CO_2_. Moreover, the carbonic anhydrase (CA) facilitates conversion of CO_2_ into H^+^ and HCO_3_^−^, meaning that CO_2_ is one of major sources of H^+^. Although CO_2_ produced in TCA cycle is converted into H^+^ and HCO_3_^−^, the amount of H^+^ produced in TCA cycle is much smaller than that produced only in glycolysis to obtain a fixed amount of ATP. In another word, from a viewpoint of requirement of the fixed amount of ATP, the amount of produced H^+^ is much smaller in cells functioning TCA cycle cooperatively with the glycolysis process compared with cells producing ATP predominantly via glycolysis but not followed by function of TCA cycle. In type 2 diabetes mellitus patients, mitochondria function decreases [17,56,121,122,123,124,125,126,127,128,129,130,131]. These observations [17,56,121,122,123,124,125,126,127,128,129,130,131] lead us to an idea that the type 2 diabetes mellitus patients produce a much larger amount of H^+^ than that produced in healthy persons keeping normal mitochondrial function. Even in cases that ‘arterial’ blood pH in type 2 diabetes mellitus patients except severe metabolic disorders stays within the normal range (7.35~7.45), the pH of interstitial fluids would be lower than 7.35.

In addition to glucose metabolism, I should describe other sources of H^+^. Another major source of H^+^ is the ketone body. For instance, beta-hydroxybutyric acid (CH_3_-CH(OH)-CH_2_-COOH), one of the most major ketone bodies (~70% of total ketone bodies), is produced by TCA cycle in liver mitochondria via oxidation of free fatty acids released from adipocytes [132]: metabolites of fatty acids in the liver provide H^+^ via a dissociating process into beta-hydroxybutyrate^−^ (CH_3_-CH(OH)-CH_2_-COO^−^) and H^+^ (CH_3_-CH(OH)-CH_2_-COOH→CH_3_-CH(OH)-CH_2_-COO^−^ + H^+^) [133]. Another major ketone body is acetoacetic acid (CH_3_-CO-CH_2_-COOH), which is converted to beta-hydroxybutyric acid (CH_3_-CH(OH)-CH_2_-COOH). These ketone bodies are synthesized in liver mitochondria only when blood glucose is not available. Under this condition, the ketone body produced in liver mitochondria is delivered to extra-hepatic tissues such as heart and skeletal muscles via blood circulation [134]. The delivered ketone bodies such as beta-hydroxybutyric acid and acetoacetic acid to muscle tissues become the source of acetyl CoA, which is a substrate producing ATP in TCA cycle in mitochondria of muscles [134]. In addition to this ketone-mediated pathway, fatty acids can be also directly converted to acetyl CoA without the ketone-mediated process; however, this direct conversion pathway of fatty acids to acetyl CoA is a minor process compared with the process via the ketone bodies synthesis. Thus, fatty acids play an important role in the ATP synthesis in mitochondria. If the energy source is only the fatty acid, a large amount of fatty acids have to be converted into ketone bodies and the amount of ketone bodies produced in liver mitochondria would exceed the mitochondria metabolizing capacity in muscles [134]. In these cases, our bodies generate an excessive amount of ketone bodies, resulting in elevation of H^+^ concentration (lowered pH) in the interstitial fluid. Low glucose use or low mitochondrial function in muscles and adipocytes leads to a condition with accumulation of excessive amounts of ketone bodies in peripheral tissues, producing a large amount of H^+^ (lowered pH). If this large amount of H^+^ produced in cells is not extruded to the extracellular space, the intracellular pH reaches a very low level, much less than 7.35. To prevent the lowered pH in the intracellular space, various H^+^ transporting systems contribute to extrusion of H^+^ into the interstitial fluid space. These processes produce the environment with a low pH value of the interstitial fluid around the peripheral metabolizing tissues including muscles.

## 5. Transporting Systems of H^+^ between the Intracellular and Interstitial Fluid Spaces

**Figure 3:**
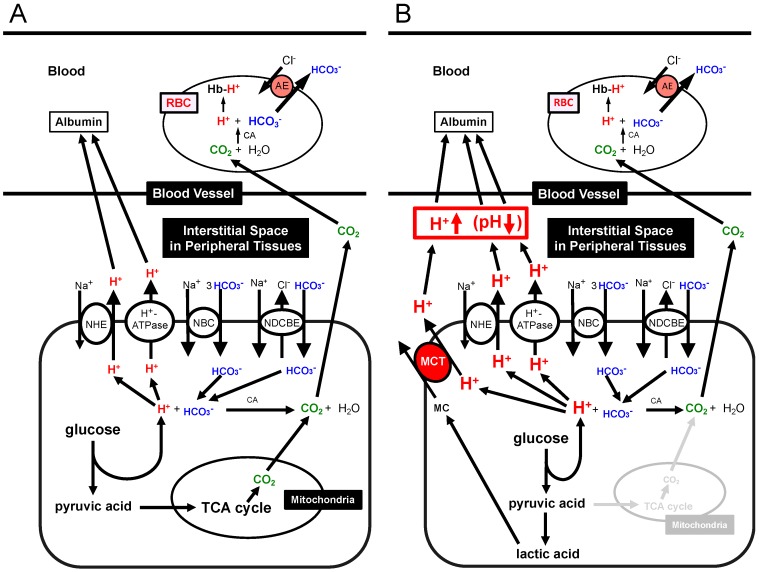
Production of H^+^ and CO_2_ and transporting systems of H^+^ and CO_2_ in peripheral tissues. (**A**) Production of H^+^ and CO_2_ and transporting systems of H^+^ and CO_2_ in peripheral tissues with ‘normal’ mitochondrial function: the glycolysis process produces H^+^ and TCA cycle generates CO_2_. (**B**) Production of H^+^ and CO_2_ and transporting systems of H^+^ and CO_2_ in peripheral tissues with ‘dysfunction’ of mitochondria. Much more amounts of H^+^ are produced via glycolysis in a case of mitochondrial dysfunction in order to produce the same amount of ATP as that with normal mitochondrial function. In a case of mitochondrial dysfunction, TCA cycle has no or little function, thus the required amount of ATP is mainly generated via glycolysis, leading to production of much more amounts of H^+^ and lactic acid than the normal case. Modified from Figure 2 in *World J Diabetes* 6(1): 125–135, 2015 [1].

For ATP synthesis, the glycolysis process produces H^+^ and the process of TCA cycle generates CO_2_. H^+^ is directly extruded via Na^+^/H^+^ exchanger (NHE) and H^+^-ATPase from intracellular to extracellular (interstitial) spaces, and binds to albumin after moving into blood (Figure 3A) [1,108,109]. Furthermore, a part of H^+^ produced at the glycolysis process is converted to CO_2_ and H_2_O consuming HCO_3_^−^ (H^+^ + HCO_3_^−^→CO_2_ + H_2_O) via a CA-facilitated process (Figure 3A) [1]. To supply HCO_3_^−^ into cells, Na^+^-driven Cl^−^/HCO_3_^−^ exchanger (NDCBE) and Na^+^-HCO_3_^−^ cotransporter (NBC) participate in uptake of HCO_3_^−^ from extracellular (interstitial) spaces (Figure 3A) [1]. Both types of CO_2_ generated from glycolysis and TCA cycle easily permeate the plasma membranes of the peripheral tissue cells and red blood cells (RBC, erythrocyte) based on high CO_2_ permeability to the plasma membrane, moving into RBC (Figure 3A) [1]. The CO_2_ moving into the intracellular space of RBC is converted into H^+^ and HCO_3_^−^ consuming H_2_O via a CA-facilitated process (Figure 3A) [1]. H^+^ produced from CO_2_ and H_2_O in RBC binds to Hb (Figure 3A) [1,108,109]. On the other hand, HCO_3_^−^ produced from CO_2_ and H_2_O in RBC is extruded to the extracellular space in the blood vessel via an exchange process with extracellular Cl^−^ by anion exchanger (AE): this exchanging step of HCO_3_^−^ extrusion and Cl^−^ uptake is so called as Cl^−^ shift (Figure 3A) [1,108,109]. Figure 3B shows the production process of H^+^ and CO_2_ and transporting systems of H^+^ and CO_2_ in peripheral tissues with ‘dysfunction of mitochondria’ (Figure 3B) [1,108,109]. A much higher amount of H^+^ are produced via glycolysis in a case of ‘mitochondrial dysfunction’ in order to produce the same amount of ATP as that with normal mitochondrial function (Figure 3B) [1,108,109]. In this case, TCA cycle has no or little function, thus the required amount of ATP is mainly synthesized via glycolysis, leading to production of much more amounts of H^+^ and lactic acid (Figure 3B) [1,108,109]. Lactic acid is produced from pyruvic acid, a substrate of TCA cycle, under an ‘anaerobic’ or ‘mitochondrial dysfunctional’ condition. (Figure 3B) [1,108,109]. This lactic acid (a monocarboxylic acid, MC) is extruded via H^+^-coupled monocarboxylate transporter (MCT) to the interstitial space, and binds to albumin after moving into blood (Figure 3B) [1,108,109]. A little part of H^+^ is converted to CO_2_ consuming HCO_3_^−^ (Figure 3B) [1,108,109]. Then, this CO_2_ moves into RBC and is converted into H^+^ and HCO_3_^−^ via a CA-facilitated process (Figure 3B) [1,108,109]. In RBC, H^+^ binds Hb, and the intracellular HCO_3_^−^ is exchanged with extracellular Cl^−^ via AE (Figure 3B) [1,108,109] As results from a large amount of H^+^ produced under mitochondrial dysfunctional conditions, the interstitial fluid pH becomes lower compared with that under the normal condition, even if albumin and Hb function as strong pH-buffering molecules in blood (Figure 3B) [1,108,109].

**Figure 4:**
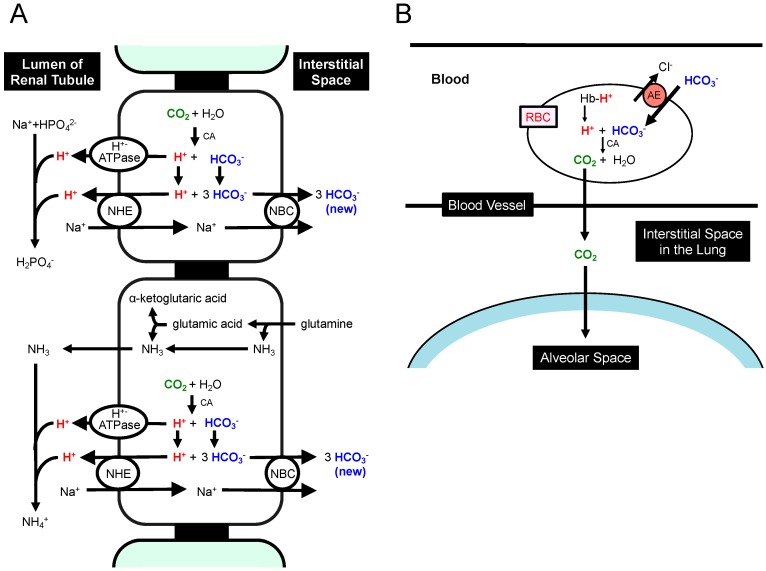
Production of HCO_3_^−^ in the kidney (**A**) and transporting systems of H^+^ and CO_2_ in the lung (**B**). (**A**) The H^+^ extrusion system into urine and new synthesis of HCO_3_^−^ in the kidney. CO_2_ is converted into H^+^ and HCO_3_^−^ via a CA-facilitated process. The H^+^ generated from CO_2_ is extruded into urine as a form of NH_4_^+^ or HPO_4_^−^ by binding to NH_3_ or HPO_4_^2−^ (NH_3_ + H^+^→NH_4_^+^: HPO_4_^2−^ + H^+^→H_2_PO_4_^−^). If CO_2_ is supplied, HCO_3_^−^ is newly generated and functions as a pH-buffering material coupled with a process of H^+^ extrusion into urine. (**B**) The extrusion system of CO_2_ in the lung. CO_2_ is converted from H^+^ and HCO_3_^−^ produced in the peripheral tissues. In the lung, the process (H^+^ + HCO_3_^−^→CO_2_ + H_2_O), which is the reversible process occurring in peripheral tissue (refer to the process in RBC described in Figure 2), occurs due to low CO_2_ circumstances in the lung. HCO_3_^−^ generated from CO_2_ in peripheral tissues is consumed to produce CO_2_, which is released to atmosphere. This means that HCO_3_^−^ generated from CO_2_ in peripheral tissues is not a net source of HCO_3_^−^ when CO_2_ is released to atmosphere in the lung. Modified from Figure 2 in *World J Diabetes* 6(1): 125–135, 2015 [1].

Lactic acid (CH_3_-CH(OH)-COOH; pKa = 3.86) is dissociated into the form of CH_3_-CH(OH)-COO^−^ + H^+^ under conditions with physiological pH (~7.40), which is much higher than pKa of lactic acid. This means that lactic acid plays a role as a source of H^+^ at normal (physiological) pH. Furthermore, other metabolites such as ketone bodies also supply H^+^. Beta-hydroxybutyric acid (CH_3_-CH(OH)-CH_2_-COOH; pKa = 4.70: one of typical ketone bodies, a metabolite of fatty acids produced in the liver) supplies H^+^ via dissociation into CH_3_-CH(OH)-CH_2_-COO^−^ and H^+^ at normal (physiological) pH. The H^+^ produced at cellular metabolisms is extruded via the following two pathways. (1) A direct form of H^+^ is extruded from the intracellular to the interstitial spaces by various types of H^+^ transporters such as Na^+^/H^+^ exchanger (NHE) and H^+^-ATPase (Figure 3A,B). (2) An indirect form of H^+^ coupled with HCO_3_^−^ (H^+^ + HCO_3_^−^→CO_2_ + H_2_O) in a CA-facilitated pathway, CO_2_, leaves from cells into the interstitial fluid space owing to high membrane permeability of CO_2_ (Figure 3A,B) [135,136,137,138,139,140]. The HCO_3_^−^ consumed in the intracellular space (Figure 3A) is transported from the interstitial space by HCO_3_^−^ transporters such as Na^+^-driven Cl^−^/HCO_3_^−^ exchanger (NDCBE) and Na^+^-HCO_3_^−^ cotransporter (NBC) (Figure 3A,B). HCO_3_^−^ produced in the kidney but not in other tissues can be the net source of HCO_3_^−^, since H^+^ generated from CO_2_ in the kidney but not in other tissues is extruded into urine (Figure 4A), while HCO_3_^−^ generated simultaneously from CO_2_ is not extruded into urine but stays inside the body. This leads to a condition that H^+^ generated from metabolites such as lactic acid reduces the intracellular concentration of HCO_3_^−^ via the CA-mediated conversion of H^+^ and HCO_3_^−^ into CO_2_ and H_2_O (H^+^ + HCO_3_^−^→CO_2_ + H_2_O) with compensatory elevation of HCO_3_^−^ uptake via NDCBE and NBC into the intracellular space (Figure 3A,B). Under a condition with an extremely large amount of H^+^ produced in cells, H^+^ is converted into CO_2_ and H_2_O by spending a large amount of HCO_3_^−^ (Figure 3B). This CO_2_ produced from H^+^ and HCO_3_^−^ via a CA-mediated process moves into RBC penetrating the plasma membrane, and in RBC this CO_2_ is again converted into H^+^ and HCO_3_^−^ via a CA-facilitated process (Figure 3B). In RBC, H^+^ binds to Hb, and anion exchanger (AE) extrudes HCO_3_^−^ from the cytosolic space to the extracellular space of RBC in blood (Figure 3B). These processes indicate that even if HCO_3_^−^ is consumed to reduce H^+^ in metabolizing cells, HCO_3_^−^ is again regenerated in RBC, meaning that HCO_3_^−^ is not consumed in peripheral tissues but plays a role as a shuttle of the H^+^ transporting system. In the lung (Figure 4B), P_CO_2__ is much lower in the lung than that in peripheral tissues. Thus, the conversion process, HCO_3_^−^ + H^+^→CO_2_ + H_2_O, occurs to produce CO_2_ in RBC (Figure 4B), and this process stimulates H^+^ release from Hb. Then, the concentration of HCO_3_^−^ in RBC reduces, and HCO_3_^−^ is incorporated into RBC via AE from the extracellular space of RBC in blood (Figure 4B). The incorporated HCO_3_^−^ is further converted to CO_2_ with H^+^ released from Hb (Figure 4B). CO_2_ produced in this process is extruded across the plasma membrane to atmosphere (Figure 4B). Under severed metabolically disordered conditions (Figure 3B), the source of HCO_3_^−^ consumed to produce CO_2_ with H^+^ in the lung (Figure 4B) is not CO_2_ as a product in TCA cycle in peripheral tissues, but HCO_3_^−^ itself originally existing in blood produced in the kidney (Figure 4A). Thus, these overall processes reduce the concentration of HCO_3_^−^ in blood under severed metabolically disordered conditions (Figure 4A), and severe overproduction of H^+^ (Figure 3B) leads us to metabolic acidosis consuming HCO_3_^−^ with pH of ‘arterial’ blood <7.35. General metabolic disorders are recognized to be the main cause of this metabolic acidosis; however mitochondrial dysfunction is recently indicated to be one of the most important disorders appearing in diabetes mellitus [17,56,121,126,127]. This mitochondrial dysfunction in diabetes mellitus is considered to be one of the main causes leading to metabolic acidosis. Furthermore, it is notable that lowered pH is also caused by H^+^ released from ketone bodies, beta-hydroxybutyric acid, and acetoacetic acid, produced in the liver via oxidation of free fatty acids originated from adipocytes [132]. Unavailability of blood glucose in muscles causes the synthesis of these ketone bodies in the liver mitochondria for ATP synthesis: ATP is synthesized from these ketone bodies as sources of acetyl CoA in TCA cycle of muscle mitochondria [134]. However, in diabetes mellitus with mitochondrial dysfunction (Figure 3B), these ketone bodies produced in the liver are not used as sources of acetyl CoA for generation of ATP via TCA cycle in dysfunctional mitochondria of muscles [134]. Thus, under these conditions in diabetes mellitus, the ketone bodies provide an excessive amount of H^+^, shifting pH to a lowered value (acidosis) compared with that with normal mitochondrial function.

Lactic acid in the intracellular space used via oxidation as a respiratory fuel is one of the useful energy sources [128]. Thus, lactic acid produced in the cytosolic space of fast muscles contributing to relatively heavy exercise under physiological conditions is transported into the interstitial (extracellular) space via monocarboxylate transporter (MCT) (Figure 3B) [141,142], and the extracellular lactic acid shuttles to oxidative tissues via the blood-delivered system [141]. Mitochondrial dysfunction in diabetes mellitus patients leads fast muscles to produce lactic acid even in cases of regular exercise without heavy muscle contraction [127,143]. In most mammalian cells, MCTs play an important role in extrusion of lactic acid and other monocarboxylic acids such pyruvic acid, beta-hydroxybutyric acid and acetoacetic acid from the cytosolic space to the extracellular space across the cellular membrane (Figure 3B) [144,145,146]. Expression of MCTs is altered in diabetes mellitus [147,148]. Since MCTs transport monocarboxylic acid coupled with H^+^, MCTs function in the process of H^+^ extrusion coupled with extrusion of monocarboxylic acid (MC) (Figure 3B). This means that MCTs play an essentially important role in balance of pH and energy in diabetes mellitus patients. The new synthesis of HCO_3_^−^ occurs only in the kidney (Figure 4A) but notother organs/tissues (Figure 4A). Therefore, under a condition with normal kidney function, the newly synthesized HCO_3_^−^ is available as an interstitial fluid pH-buffering material. However, under a pathophysiological condition with abnormal kidney function, no or little newly synthesized HCO_3_^−^ is available as an interstitial pH-buffering material, resulting in lowered pH conditions in the interstitial fluid.

On the other hand, it is suggested that obesity-induced inflammation engenders insulin resistance and type 2 diabetes mellitus. However, the mechanism is unclear [149]. Protein tyrosine phosphatase receptor gamma (PTPR-gamma) is indicated as a key factor linking the obesity-induced inflammation and insulin resistance in type 2 diabetes mellitus [149]. Although PTPR-gamma is a key factor causing insulin resistance in type 2 diabetes mellitus, inflammation is well known to be associated with lowered interstitial pH, which would cause insulin resistance.

## 6. Roles of Abnormal Interstitial Fluid pH in Diabetes Mellitus

Insulin is a key hormone for homeostasis and use of blood glucose in the body. The glucose uptake into skeletal muscles is stimulated by insulin via enhancement of glucose transporter 4 (GLUT4) translocation from the cytosolic store site to the plasma membrane, decreasing the blood glucose level [150,151]. The first step for insulin to show its stimulatory action on glucose uptake is the insulin binding to its receptor located on the plasma membrane. Then, tyrosine residues of the receptor are immediately auto-phosphorylated, subsequently followed by phosphorylation of tyrosine residues of insulin receptor substrate-1 (IRS-1). IRS-1 phosphorylation induces activation (phosphorylation) of phosphoinositide 3-kinase (PI3K), which catalyzes 3′ phosphorylation of phosphatidylinositol 4,5-diphosphate (PIP2), leading to activation of Akt. This PI3K/Akt-mediated signaling in the insulin-induced down-stream pathway stimulates translocation of GLUT4 to the plasma membrane from the cytosolic store site, elevating glucose uptake into skeletal muscles. Insulin resistance is recognized as dysfunction of this insulin signal transduction in glucose uptake into skeletal muscles in type 2 diabetes mellitus [152]. The pH value of interstitial fluids is lower in Otsuka Long-Evans Tokushima Fatty (OLETF) rats, a model of type 2 diabetes mellitus, than that in normal ones [113]. Metabolic acidosis is reported to induce insulin resistance [153], although the molecular mechanism is not yet fully understood. Reports from several laboratories indicate that acidosis caused by organic acids would develop insulin resistance in its early stages [110,113,153,154,155,156,157]. Furthermore, it has been reported that insulin sensitivity is negatively correlated with body weight [158]. The value of 24-h urine pH in persons with metabolic syndrome is significantly lower than that in healthy persons [156]. Persons with metabolic acidosis showing larger anion gap associated with lower serum HCO_3_^−^ indicate less insulin sensitivity (higher insulin resistance) [159]: low serum HCO_3_^−^ means that the body produces a large amount of H^+^, which would consume serum HCO_3_^−^ for pH homeostasis. The interstitial fluids around brain hippocampus and metabolic tissues in OLETF rats show lower pH values compared with the normal one [2,113] (Figure 2). Although we have no direct evidence on the molecular mechanism lowering pH of interstitial fluids, these phenomena would be caused by dysfunction or hypo-function of mitochondria in diabetes mellitus [17,56,126,127]. The pH-buffering capacity of the interstitial fluid is much lower compared with that in the blood and in the cytosol (Figure 1 and Figure 3), meaning that interstitial fluid pH in metabolic tissues is variable depending on metabolic conditions. One of the most serious problems in glucose metabolism caused by the lowered pH value of the interstitial fluid [1,108,109,110,113,160,161,162,163,164,165,166] is occurrence of the insulin resistance [1,108,109,110,113]. The lowered interstitial fluid (extracellular) pH diminishes insulin action on glucose uptake in rat skeletal model cells [110,167]. The lowered interstitial (extracellular) fluid pH decreases insulin binding affinity to its receptor associated with lowered insulin receptor phosphorylation (activation) with no change in total or surface expression of insulin receptors in skeletal muscles due to some conformational changes in the receptor and insulin [110]. This diminution of the insulin binding affinity caused by the lowered interstitial pH attenuates Akt phosphorylation (activity), a down-stream molecule in the insulin signaling pathway [110], resulting in a low level in the insulin-stimulated glucose uptake [110]. These observations indicate that the lowered interstitial fluid pH is one of the most important factors developing insulin resistance.

## 7. Roles of Foods Containing Weak Organic Acids and Carboxylate Transporters in Improvement of Low Interstitial Fluid pH

**Figure 5:**
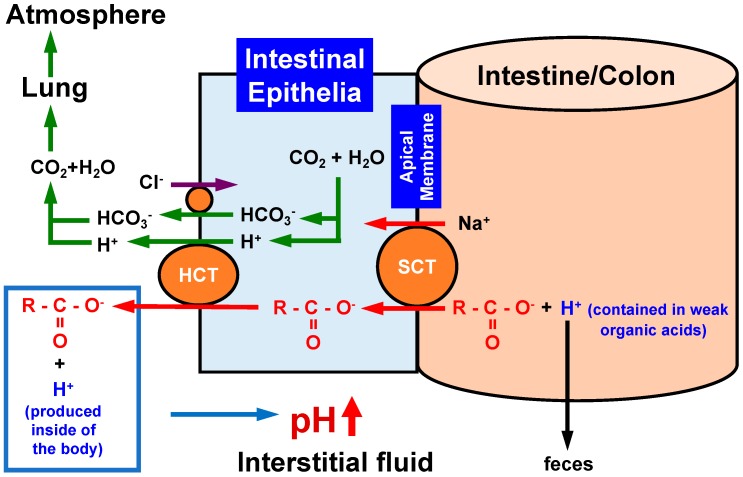
Action of oral intake of foods containing weak organic acids with carboxyl groups on pH regulation of the interstitial fluid. When we intake the weak organic ‘acid’ containing a carboxyl part (R-COO^−^), only the carboxyl part (R-COO^−^) is absorbed via sodium-coupled carboxylate transporters (SCT) expressed in the apical membrane of the intestine. Incorporated carboxyl groups are transported from the intracellular space to the extracellular space (the interstitial space) of epithelial cells via H^+^-coupled carboxylate transporters (HCT). H^+^ contained in weak organic acids is not absorbed in the intestine, but is excreted into feces. Thus, weak organic acids behave as ‘bases’ by combing with H^+^ produced in the body, elevating pH. This means that weak organic acids play a role as pH buffers in the interstitial fluid.

**Figure 6:**
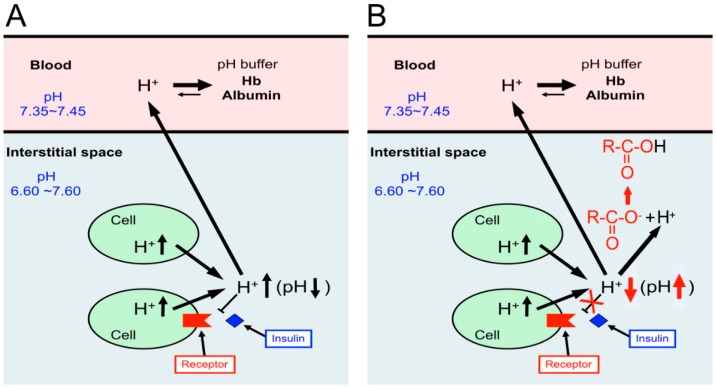
Insulin resistance caused by lowered interstitial fluid pH in diabetes mellitus (**A**), and elevation of interstitial fluid pH and improvement of insulin resistance by intake of foods containing weak organic acids with carboxylic groups (R-COO^−^) (**B**). (**A**) The lowered value of interstitial fluid pH diminishes the insulin binding affinity to its receptor, causing the insulin resistance in diabetes mellitus. (**B**) Elevation of interstitial fluid pH by intake of foods containing weak organic acids with carboxylic groups (R-COO^−^) increases the binding affinity of insulin to its receptor, improving the insulin resistance.

A recent review article [168] introduces the role of excessive energy intake and reduced energy expenditure in development of insulin resistance in diabetes mellitus based on the crosstalk among various nutrients. However, no evidence or discussion is available on the role of the nutrients in pH regulation in the body. As shown in Figure 1 and Figure 3, the interstitial fluid has little pH buffer compared with blood. Figure 5 shows the relationship between the intake of carboxylate, one of weak organic acids commonly contained in foods, and its pH buffering action [115,116]. When we take weak organic acids such as acetic acid (CH_3_COOH: pKa = 4.76), propionic acid (CH_3_CH_2_COOH: pKa = 4.88), palmitic acid (CH_3_(CH_2_)_14_COOH: pKa = 4.95), citric acid ((CH_2_COOH)_2_COOH: pKa = 3.13, 4.76 and 6.40), which are commonly contained in foods. In general, the weak organic acids have values of pKa much less than the physiological pH value, 7.4, of ‘arterial’ blood and the interstitial fluid [115,116]. This means that these acids exist as ionized forms (R-COO^−^ + H^+^) under physiological and even pathophysiological acidic conditions. An interesting point is the absorbing mechanism of these acids in the intestine. Only R-COO^−^, but not H^+^, of these weak organic acids is absorbed via Na^+^-coupled carboxylate transporters such as sodium-dependent monocarboxylate transporter 1 (SMCT1) and sodium-dependent dicarboxylate transporter (NaDC1) expressed at the apical membrane of the intestine (Figure 5) [169,170,171,172]. The H^+^ contained in the weak organic acid is not absorbed into the body, but excreted to the outside of the body as a content of the feces (Figure 5). The absorbed R-COO^−^ into epithelial cells of the intestine is transported to the interstitial fluid space via H^+^-dependent carboxylate transporters (HCT) including monocarboxylate transporter (MCT1) (Figure 5). Some parts of R-COO^−^ transported into the interstitial fluid space bind with H^+^ produced in the metabolic process of the body, elevating the interstitial fluid pH (lowering the H^+^ concentration). Namely, the absorbed R-COO^−^ behaves as a ‘base’. A report [173] indicates that intake of omega-3 polyunsaturated fatty acids (weak organic acids) improves (decreases) high-fat diet-induced high blood glucose, glucose uptake, glucose oxidation and glycogen synthesis. This report [173] also shows that intake of omega-3 polyunsaturated fatty acids enhances the insulin signals, Akt and GSK3-beta, indicating that intake of omega-3 polyunsaturated fatty acids improves the high-fat diet-induced insulin resistance. This report [173] concludes that the action of omega-3 polyunsaturated fatty acids on insulin resistance and levels of blood glucose and insulin is mediated via improvement of skeletal muscle mitochondrial function. However, it is possible that this action of omega-3 polyunsaturated fatty acids [173] appears via elevation of interstitial fluid pH (Figure 5). Indeed, our experimental observations indicate that citrate intake improves serum glucose levels in diabetes mellitus [174] associated with elevation of interstitial fluid pH (Figure 6) [174]. Furthermore, products by honey bees, propolis extracts, improve insulin resistance via elevation of the interstitial fluid pH [113]. Some traditional medicinal compounds also improve insulin resistance via elevation of the interstitial fluid pH [175]. The food intake with weak organic acids elevating the interstitial fluid pH (Figure 5) improves the insulin resistance, which appears in diabetes mellitus with lowered interstitial fluid pH values (Figure 6A), by increasing the insulin sensitivity (Figure 6B), although the food intake with weak organic acids has little effects on the pH value of arterial blood with strong pH buffers such as Hb and albumin (Figure 6).

## 8. Proposal of Molecular Mechanisms of Diabetes Mellitus Occurrence Caused by High Salt Intake

**Figure 7:**
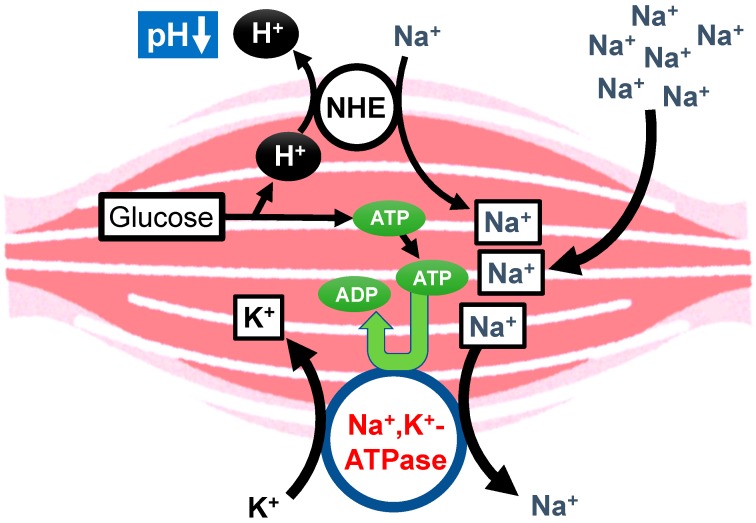
Proposal of the molecular mechanism of the high salt intake-induced development of diabetes mellitus. High salt intake produces a lot of H^+^, since high salt intake leads to consumption of ATP required for the Na^+^, K^+^-ATPase to extrude the high leveled intracellular Na^+^ caused by high salt intake in cells such as muscles.

High salt intake has been indicated to develop type 2 diabetes mellitus [3]. However, the molecular mechanism of the high salt intake-induced development of type 2 diabetes mellitus is still unclear. High salt intake would produce a lot of H^+^ associated with production of ATP, since high salt intake leads to consumption of ATP required for the Na^+^, K^+^-ATPase to extrude the intracellular Na^+^ at a high level caused by high salt intake in cells such as muscles (Figure 7). Thus, high salt intake would lower the interstitial fluid pH, resulting in insulin resistance and diabetes mellitus, although more experimental evidence is required to prove this proposal mechanism.

## 9. Conclusions

**Figure 8:**
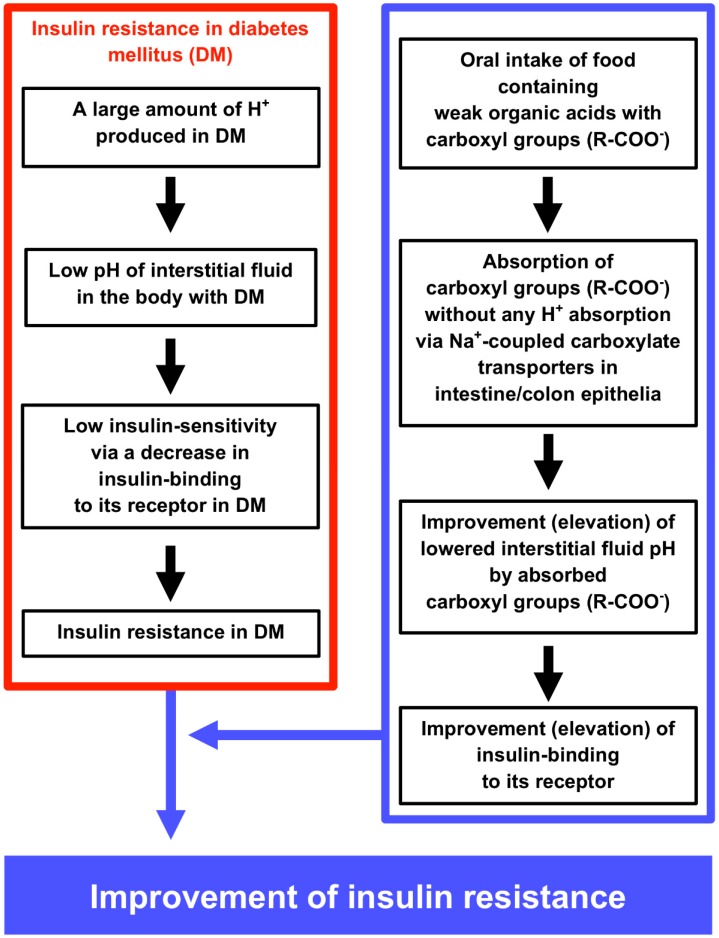
The molecular mechanism causing low interstitial fluid pH and insulin resistance in diabetes mellitus (DM) (left column), and the improvement of insulin resistance by oral intake of foods containing weak organic acids with carboxyl groups (right column). The left column inside of the red square indicates the molecular mechanism causing DM and insulin resistance via lowering interstitial fluid pH by producing a lot of H^+^. The right column inside of the blue square indicates the molecular mechanism of oral intake of foods containing weak organic acids with carboxyl groups improving lowered pH of interstitial fluids, insulin binding and insulin resistance under the DM condition.

Interstitial fluids have limited pH-buffering capacity. Due to this limitation of the interstitial fluid pH-buffering capacity, over production of acid metabolites leads the interstitial fluid pH to a lowered value even when the pH values of the cytosolic space and ‘arterial’ blood remain within the normal range. The most serious problem of lowered pH of interstitial fluids is that lowered pH of interstitial fluids causes insulin resistance by diminishing insulin binding affinity to its receptor. Acidic circumstances caused by mitochondrial dysfunction observed in type 2 diabetes mellitus lead to insulin resistance. Food intake with weak organic acids improves (elevates) the interstitial fluid pH, which is lower in diabetes mellitus than normal one. This food intake-induced elevation of the interstitial fluid pH improves the insulin resistance by increasing the insulin binding to its receptor (elevation of the insulin sensitivity). The summary of this review article is shown in Figure 8.

## Abbreviations

AE	anion exchanger
CA	carbonic anhydrase
DM	diabetes mellitus
GLP	glucagon-like peptide
GLUT4	glucose transporter 4
[H^+^]	H^+^ concentration
Hb	hemoglobin
HCT	H^+^-coupled carboxylate transporter
IDF	International Diabetes Federation
IRS-1	insulin receptor substrate-1
MC	monocarboxylic acid
MCT	monocarboxylate transporter
NBC	Na^+^-HCO_3_^−^ cotransporter
NDCBE	Na^+^-driven Cl^−^/HCO_3_^−^ exchanger
NHE	Na^+^/H^+^ exchanger
OLETF	Otsuka Long-Evans Tokushima Fatty
PIP2	phosphatidylinositol 4,5-diphosphate
PPD	dipeptidyl-peptidase
PTPR	protein tyrosine phosphatase receptor
RBC	red blood cells
SCT	sodium-coupled carboxylate transporters
SGLT2	sodium-glucose cotransporter 2
